# Helping patients with chronic diseases quit smoking by understanding their risk perception, behaviour, and smoking-related attitudes

**DOI:** 10.1371/journal.pone.0284690

**Published:** 2023-04-20

**Authors:** Laurie Long Kwan Ho, William Ho Cheung Li, Ankie Tan Cheung

**Affiliations:** The Nethersole School of Nursing, The Chinese University of Hong Kong, Hong Kong, Hong Kong SAR; The University of Leeds, UNITED KINGDOM

## Abstract

Continued smoking among patients with chronic diseases detrimentally affects their health and treatment outcomes. However, a majority of smokers with chronic diseases appear to have no intention to quit. Understanding the needs and concerns of this population is a crucial step in facilitating the design of an appropriate smoking cessation intervention. This study aimed to understand the risk perception, behaviours, attitudes, and experiences related to smoking and smoking cessation among patients with chronic diseases, including cardiovascular diseases, chronic respiratory diseases, and/or diabetes in Hong Kong. Individual semi-structured interviews with smokers with chronic diseases (n = 30) were conducted from May to July 2021. The methods and results are reported according to the COREQ. Four themes were generated: (1) perceptions of the association between chronic diseases and smoking/smoking cessation; (2) perceptions of the health/disease status; (3) quitting smoking is not the first priority; and (4) perceived barriers to quitting smoking. This study addressed a gap in the literature by gathering data concerning the perspectives of smokers with chronic diseases on smoking and smoking cessation. The deficit of knowledge among smokers with chronic diseases warrants the reinforcement of health education targeting this population. Our findings indicate the need for further efforts in designing appropriate smoking cessation interventions targeting smokers with chronic diseases, which will match the needs and concerns identified in this study.

## Introduction

Chronic diseases (also known as noncommunicable diseases), including cancer, cardiovascular diseases, chronic respiratory diseases, and diabetes mellitus, account for approximately 41 million deaths (71% of all deaths) worldwide annually [1]. It is estimated that chronic diseases will be responsible for approximately 55 million deaths annually by 2030 unless much more ambitious interventions and policies are implemented [2]. In Hong Kong, chronic diseases accounted for approximately 55% of all registered deaths in 2016, compounded by the aging population, which places a heavy burden on the health care system [3].

A large, convincing body of literature suggests that smoking plays a causal role in the development of certain chronic diseases [4]. Smoking is the leading preventable cause of morbidity and mortality, accounting for 8 million deaths annually worldwide [5]. The adverse health effects of smoking are more apparent in adults aged >30 years, with substantial increases in the morbidity and mortality rates associated with chronic diseases from early middle age [6]. Continuation of smoking among patients with chronic diseases may also detrimentally affect health and treatment outcomes, increasing the risk of disease progression or recurrence and thus elevating the risk of mortality and reducing the efficacy of treatment [7–9]. The prevalence of smoking ranged from 15% to 26% among patients with chronic disease [10]. According to the World Health Organization [11], 14% of all deaths from chronic diseases were attributable to smoking.

A large amount of data shows that smoking cessation confers immediate health benefits (e.g., decreases in the heart rate and blood pressure) that only increase over subsequent years [12, 13]. For patients with chronic disease, smoking cessation decreases the risk of disease progression or recurrence, increases the chances of survival, and improves treatment efficacy; thus, these patients represent a key target group for smoking cessation interventions [7–9].

Nevertheless, a majority of smokers with chronic diseases tend to share certain characteristics, including a long smoking history, strong nicotine dependency, and no intention to quit [14–16]. Chronic conditions were also found to predict low motivation to quit as well as the likelihood of smoking relapse [17, 18]. Several studies have indicated that the majority of Hong Kong Chinese with chronic diseases who smoke were in the precontemplation stage (i.e., no intention to quit) [19]; of these, 68% had cardiovascular disease [14], 70% had diabetes mellitus [16], and 73% had cancer [15]. A recent systematic review involving 10 interventional trials targeting adult smokers with chronic diseases found that the participant selection criteria in most studies excluded unmotivated smokers, indicating that most existing interventions and studies do not include the majority of smokers with chronic diseases [10]. This finding underscores the critical need for developing and evaluating appropriate smoking cessation interventions to promote smoking cessation targeting this population.

Understanding the needs and concerns of smokers with chronic diseases is a crucial step in facilitating the design of an appropriate smoking cessation intervention; however, a review of relevant literature indicated the lack of qualitative evidence on chronic disease patients’ perceptions and experiences regarding smoking/smoking cessation. Studies performed using a qualitative approach can yield an in-depth description of the actual concerns of this population and how their feelings are shaped by their culture [20]. Therefore, this study aimed to understand the risk perception, behaviours, attitudes, and smoking cessation-related experiences of smokers with chronic diseases.

## Materials and methods

### Study design

A descriptive qualitative research design was used. Smokers with chronic diseases were convenience sampled from May to July 2021. The methods and results are reported according to the Consolidated Criteria for Reporting Qualitative Studies (COREQ) [21] (See S1 Appendix). Data saturation was used to determine the sample size of this study (i.e., no additional data emerged in data analysis), which was reached after interviewing 30 smokers; a 100% response rate was achieved. All interviews were conducted in Cantonese.

### Participants

All eligible patients with chronic diseases who were attending a medical follow-up at one of the largest general outpatient clinics at a public acute-care hospital in Hong Kong were invited to participate in the study. The inclusion criteria were as follows: (1) patients aged ≥18 years; (2) patients who smoked at least one cigarette per day over the previous 3 months; (3) patients who had been diagnosed with at least one chronic disease; and (4) patients who were able to speak Cantonese. The exclusion criteria were as follows: (1) patients with mental or cognitive impairment or communication problems or (2) those with unstable physical conditions, as indicated by their physician.

### Data collection

A research assistant approached the participants who were attending a medical follow-up and invited them to attend an individual semi-structured interview. Written informed consent was obtained prior to the study. The interviews were audio-taped and lasted for 30−40 min. All of the interviews were conducted in a private consultation room by two qualified nurses who had extensive experience in conducting qualitative interviews and had received training from a professor with expertise in smoking cessation and chronic diseases. One nurse worked as an interviewer and elicited the participants’ feelings/thoughts freely and honestly. The other nurse played the role of an observer and sought to detect the participants’ nonverbal cues, including facial expressions and body gestures. Field notes were taken throughout the interviews.

A semi-structured interview guide was developed by a group of qualitative and smoking cessation/chronic disease research experts; this group included a professor, an associate professor, an assistant professor, and two postdoctoral fellows. The interview guide was further assessed for relevancy and appropriateness by a senior medical officer with 20 years of experience in treating patients with chronic diseases and nurse counsellors with >5 years of experience in providing counselling related to smoking cessation.

The interviews began with a broad and open question, for example, ‘Can you share something about your chronic disease(s)?’ This question was followed by nondirective questions related to the participant’s heath status (e.g., ‘What do you think about your health condition?’ or ‘How do you evaluate your health status?’) and smoking habits (e.g., ‘What are your perceptions of the relationship between smoking and your chronic disease(s)?’). Different probing techniques (e.g., ‘Can you give me some examples?’) were applied throughout the interviews to elicit detailed and comprehensive information.

### Ethical considerations

The study was approved by the Institutional Review Board of the University of Hong Kong/Hospital Authority Hong Kong West Cluster (UW19-117). Written informed consent was obtained from the participants after they were informed about the purpose of the study and provided their consent to participate. Data confidentiality was assured, and the participants were informed that their participation was completely voluntary. The participants were also informed that they could withdraw from the study at any time without any negative consequences.

### Data analysis

A thematic analysis approach was used analyse qualitative data [22]. To accurately capture the contents of the dialogues and physical expressions that took place during the interview, all recordings were transcribed verbatim into Cantonese immediately after each interview. Moreover, important quotes relevant to the emerging themes were identified and translated into English for the purpose of reporting. The research assistant anonymised all data which might include identifying information of participants and identified them with a participant ID. Two researchers independently analysed the data and performed open coding on all transcripts to identify statements that were relevant to the phenomena under investigation. To improve objectivity and reduce personal bias, the two researchers recorded their data analysis procedures and compared their results to ensure stability and consistency of the findings. Codes that were common across transcripts were then grouped into categories and themes after examining their similarities. By organising all of the themes, a full and inclusive description of the phenomena emerged. Nonverbal behaviours and interactions from field notes provided additional details about the participants’ feelings, which further aided the data analysis.

### Rigour

The quality and rigour of the qualitative study in terms of its credibility, transferability, dependability, and confirmability were ensured using several strategies. The credibility of the study was enhanced by adopting triangulation strategies, including taking field notes throughout the interviews to capture any supplementary nonverbal cues and involving two researchers for data analysis [23]. By validating results with the participants, member-checking was also performed to enhance the credibility [23]. In addition, interview privacy was ensured by offering a safe and secure environment for the participants. The participants were assured of confidentiality to allow them to express their feelings and ideas freely and honestly.

Transferability was achieved by identifying similarities to the findings of other studies and was enhanced by using direct quotations of the participants and explicit descriptions of their experiences. Dependability was demonstrated using stepwise replication, which involved two researchers analysing the data independently and then comparing their findings to ensure stability and consistency [24]. Moreover, for consistency, all interviews were conducted by the same researchers. Confirmability was improved by reflecting on the data analysis procedure, which involved the two researchers recording the process of data analysis and periodically reflecting on it to maintain their objectivity [24]. Research team meetings were also held at regular intervals to monitor the data analysis process and/or to manage any divergence of opinions.

## Results

### Participant characteristics

A total of 101 patients from the general outpatient clinic were assessed for eligibility; 46 patients were found to be eligible, 30 of whom were approached and agreed to participate in the study by providing informed consent. The participants included 28 men and 2 women with a mean age of 54.6 years (SD = 10.6). The participants’ demographic and clinical characteristics are shown in Table 1. Of the 30 participants, 14 (46.7%) had multiple chronic conditions; their mean number of smoking years was 28.2 (SD = 9.06), and 63.3% (19 of 30) of the participants consumed >20 cigarettes each day. Four themes and nine subthemes were identified from the interviews. A summary of themes and subthemes is presented in Table 2.

**Table 1:** Baseline characteristics (N = 30).

Variable	Frequency (%)
**Age**, mean (SD), years	54.6 (10.6)
**Sex**	
Male	28 (93.3)
Female	2 (6.7)
**Educational attainment**	
Primary or below	4 (13.3)
Secondary	24 (80.0)
Tertiary	2 (6.7)
**Employment status**	
Employed	26 (86.7)
Unemployed or retired	4 (13.3)
**Diagnosis**	
Cardiovascular diseases	9 (30.0)
Chronic respiratory diseases	2 (6.7)
Diabetes	5 (16.7)
Multiple chronic conditions^a^	14 (46.7)
**Years of smoking**, mean (SD), years	28.2 (9.06)
**Daily cigarette consumption**	
1–10	3 (10.0)
11–20	8 (26.7)
21–30	16 (53.3)
>30	3 (10.0)

^a^ Multiple chronic conditions: two or more concurrent chronic diseases.

**Table 2:** Themes and subthemes from the semi-structured interviews.

Themes	Subthemes
1. False perceptions/knowledge of the association between chronic diseases and smoking/smoking cessation	1.1 Smoking would not cause chronic diseases, except lung-related ones
	1.2 Smoking cessation would not have positive impact on their health and existing chronic diseases
2. Perceptions of health/disease status	2.1 Self-evaluated as healthy despite chronic diseases
	2.2 Chronic disease is a necessary stage after middle age
3. Quitting smoking is not the first priority	3.1 Changing other unhealthy habits first
	3.2 Extra energy for tackling issues related to the pandemic
4. Unawareness regarding available smoking cessation services	/

### Theme 1: False perceptions/knowledge of the association between chronic diseases and smoking/smoking cessation

The theme ‘false perceptions/knowledge of the association between chronic diseases and smoking’ was generated and two subthemes were identified.

#### Subtheme 1.1 Smoking would not cause chronic diseases, except lung-related ones

Although all participants could recognise the causal relationship between smoking and lung disease, half of them were unaware of the relationship between smoking and other chronic diseases. They shared the mistaken belief that chronic diseases other than lung-related ones were caused solely by ageing and not by smoking. They were unable to establish a connection between smoking and chronic diseases, owing to a lack of knowledge about the development of these diseases.

‘I have smoked for over 30 years. If smoking could cause hypertension or diabetes, I would be diagnosed when I was young. Health problems are unavoidable when we are getting older regardless of whether we are smoking or not’. *(Participant 04)*

‘I know smoking is no good for health and causes “black lung.” However, I don’t think smoking is the main cause of chronic disease because many people, including my wife and friends who never smoke, also suffer from high blood pressure or other chronic diseases’. *(Participant 10)*

#### Subtheme 1.2 Smoking cessation would not have positive impact on their health and existing chronic diseases

Most participants did not believe that quitting smoking would have a positive impact on their existing chronic diseases; they genuinely thought that their chronic diseases had been present for a long time and would not improve even if they stop smoking. Most of them were even unaware that continuing to smoke could have a negative impact on treatment outcomes or worsen disease progression. Instead, they shared the false belief that their body had already adapted to the presence of nicotine. They believed that their body could not function well without nicotine and that quitting smoking would kill them.

‘Each inch of my body has already been occupied by nicotine. I can feel that my body could not function well if I stopped smoking’. *(Participant 07)*

‘One of my mainland relatives had smoked for over 30 years, just like me. His daughter asked him to quit smoking last year and he [is] dead this year. You should believe that people like me who have smoked for so long cannot live without smoking, especially [as] I have several diseases. My body cannot afford such a big change’. *(Participant 10)*

‘I already have a chronic disease and it is too late to quit smoking. Even if I quit smoking now, I don’t see any benefit to my existing disease’. *(Participant 15)*

‘I know smoking causes diseases, but as far as I know, smoking has no effect on my existing diseases. I have diabetes and high blood pressure; I don’t think my blood sugar and blood pressure rise when I smoke’. *(Participant 24)*

### Theme 2: Perceptions of health/disease status

The theme ‘perception of health/disease status’ was evident in the comments of participants, and two subthemes were identified accordingly.

#### Subtheme 2.1 Self-evaluated as healthy despite chronic diseases

Most participants evaluated their health conditions as healthy and believed that they had proper control of their chronic diseases. Some of them stated that they had already changed a part of their daily habits owing to their chronic disease, and that their health had improved to the point where they felt it was unnecessary for them to quit smoking at this time. Some of them also pointed that it would be too challenging for them to quit smoking while also attempting to change their other daily habits. They mentioned that it was extremely difficult to overcome withdrawal symptoms and/or cigarette cravings, given that they had smoked for so many years.

‘Although I need to take medications regularly, I usually do exercises, and I eat vegetables every day. I think I am very healthy’. *(Participant 01)*

‘Nurses told me that my blood pressure is just higher than normal range a little bit. I think everything is fine and under my control. So why [do] I need to quit smoking now?’ *(Participant 09)*

‘I have actually changed a lot of my unhealthy habits because of my diseases. I have tried to eat vegetables every night or do exercises once a week. I have already paid a lot of afford on these changes and I do not have additional energy to quit smoking at this moment’. *(Participant 11)*

‘I tried to quit but failed. I tried to chew gum or drink water or any strategies that nurses taught me but all of these did not work. It seems like giving up a friend who has accompanied me for over 30 years. It is really too hard for me’. *(Participant 21)*

#### Subtheme 2.2 Chronic disease is a necessary stage after middle age

A majority of the participants believed that it was common to have chronic diseases after middle age. They were unable to recognise the link between smoking and the development of chronic diseases, leading to the false notion that smoking is unrelated to their chronic diseases. They even considered having chronic diseases to be a necessary stage in their lives and thought that they could not prevent it even if they quit smoking.

‘Having diseases becomes normal when people are getting older. It is not a big deal. I have already lived so long and why do I still need to bother quitting smoking? Everyone has their own fate. If I die, then die’. *(Participant 02)*

‘Everyone in my age must have one or two health problems. I don’t think I need to quit smoking because of these problems’. *(Participant 06)*

### Theme 3: Quitting smoking is not the first priority

The third theme, ‘quitting smoking is not the first priority,’ was generated in interviews, and two subthemes were identified accordingly.

#### Subtheme 3.1: Changing other unhealthy habits first

Most participants thought that they had many other unhealthy habits that they would need to modify before they thought to quit smoking. They would like to change other unhealthy habits first and prioritise quitting smoking later. Most of the participants lacked motivation to quit smoking and placed other unhealthy habits ahead of smoking.

‘I know I need to quit smoking someday, but just not now. If you let me choose, I will try to do more exercises or eat more healthy food first, quit[ting] smoking can be later’. *(Participant 05)*

‘Regarding to change my unhealthy habits, smoking is not the only choice. I would like to put quitting smoking at the last because I have no confidence to do it well now’. *(Participant 11)*

#### Subtheme 3.2: Extra energy for tackling issues related to the COVID-19 pandemic

A majority of the participants admitted that they had a tough time during the pandemic. They needed extra energy to handle different issues regarding the pandemic, such as inadequate medication storage or delayed medical follow-ups owing to restricted hospital services. Some participants also expressed their worries regarding their health conditions because they were scared of the high risk of mortality if they contracted the virus. They mentioned that they did not have the extra time/energy to think about quitting smoking during this critical period. Instead, they even considered smoking as a coping strategy for stress relief. They were, however, unaware of the risk of transmission of the COVID-19 virus or even the risk of mortality associated with tobacco use. Most of them mistakenly believe that smoking is not quite as dangerous as the virus that kills them, and they even use tobacco to cope with their negative emotions. The majority of participants demonstrated a lack of knowledge in understanding the hazards of smoking and incorrectly perceived the risks posed by smoking.

‘The world is changing and I have never been experienced such a pandemic in the past. It would not be a good time to discuss about quitting since I have a lot of stuff to think about’. *(Participant 17)*

‘Although my medications are almost running out, I am worried that I will be infected if I go to hospitals to have the medical follow-ups. I seldom go out since the pandemic and smoking [has] become my only pleasure at home’. *(Participant 30)*

### Theme 4: Unawareness regarding available smoking cessation services

A majority of the participants mentioned that they were unaware of existing smoking cessation services and that they did not ever want to join such services. They mentioned that doctors or nurses seldom provided them support or professional advice to help them quit smoking, particularly to overcome withdrawal symptoms or cigarette cravings.

‘I do not really know about the existing services for quitting smoking; however, even I know that I am not interested in them’. *(Participant 13)*

‘You know, doctors and nurses are very busy. The consultation sessions usually last for about five to ten minutes. They won’t discuss too much about smoking cessation with me, not even how to quit’. *(Participant 22)*

‘I often see nurses working in hospitals. I hope that they can talk to me, and give me some advice and support for quitting smoking. However, it seems that they are very busy doing so’. *(Participant 28)*

## Discussion

This study has addressed an under-researched topic and served to answer a necessary question concerning risk perceptions and experience related to smoking/smoking cessation among Hong Kong Chinese with chronic diseases who smoke. Using a design of individualised semi-structured interviews, this study collected in-depth qualitative data and has yielded a dense description from the participants’ perspective.

Most of the participants in this study were men; this might reflect the lower smoking prevalence among women than men in Hong Kong. In 2020, a local population-based survey showed that the prevalence of daily cigarette smoking was 18.1% and 3.2% among men and women, respectively [25]. Given that men were more likely than women to engage in risky behaviours, gender differences may explain why most participants were reluctant or unprepared to quit smoking [26]. Consistent with the patients with chronic diseases in previous studies, the participants in the present study had a long smoking history and were heavy smokers [14, 15]. Moreover, most participants suffered from multiple chronic diseases. These clinical and smoking-related characteristics of the participants might explain the findings of this study; participants tended to underestimate the negative effects of smoking on their health and/or overestimate the difficulty of overcoming withdrawal symptoms [17, 27].

Our findings revealed that most smokers with chronic diseases overlooked the importance of quitting smoking due to a lack of knowledge about the correlation between smoking and the development of chronic diseases, preferring to modify other unhealthy habits first. Lack of knowledge in this area also resulted in a low motivation to quit because they wrongfully believed that smoking was not as dangerous as the viruses that would kill them. These findings are consistent with previous research that found smokers receiving medical care with low motivation to quit had low perceived vulnerability to the development of smoking-related disease [28]. In addition, the results are also in line with previous findings that smokers who perceived themselves as having a low risk in developing smoking-related disease (i.e., a lack of knowledge about the correlation between smoking and the development of chronic diseases) were less likely to quit smoking [29]. Thus, our study showed that the majority of smokers with chronic diseases lacked motivation and were unaware of the links between smoking and the development of their chronic diseases as well as the advantages of quitting smoking for the advancement of those diseases. The findings could assist healthcare professionals to predict the likelihood of smoking cessation in these patients and raise public attention to insufficient or ineffective health education regarding the hazard of smoking.

Notably, some of the participants thought that they were not able to change their fate, including the diagnosis of their diseases or even death. They believed that they could not prevent diseases or death even if they quit smoking. Their interpretation regarding diseases or death could be explained by the philosophical doctrines of the Chinese culture, namely Taoism and Fatalism, which emphasise belief in fate and destiny [30]. Chinese people tend to believe that there is a force beyond their control that dominates their fate, resulting in their inability to change their fate.

This study highlighted the main factors/barriers that impede the motivation of patients with chronic diseases to quit smoking. These findings may guide health care professionals, who play a prominent role in raising the issue of smoking cessation with patients, to design appropriate smoking cessation interventions for patients with chronic diseases. For instance, health care professionals could promote changes in the lifestyles (not smoking related) of smokers with chronic diseases at first, which might increase their readiness to comply with a larger request (e.g., quitting smoking) after a small successful step, based on the foot-in-the-door technique [31]. Health care professionals need to be proactive in introducing and promoting smoking cessation services among patients with chronic diseases, given that the study findings showed that most participants were unaware of such services and had low motivation to join such programs. Professional advice on smoking cessation is warranted, especially because our findings showed that most participants did not receive any such advice. Multidisciplinary collaboration should also be established to evaluate the existing smoking cessation services and optimise services that really meet the needs of smokers with chronic diseases.

The WHO highlighted that nurses and other health care professionals are crucial advocates of smoking cessation by providing professional advice and guidance and by responding to patients’ questions regarding smoking and associated health issues [32]. Nevertheless, most health care professionals have not received formal training on smoking cessation and thus feel incompetent to actually provide such services [33, 34]. Appropriate advanced training is essential for health care professionals to learn and implement advanced practices in smoking cessation interventions, including brief interventions targeting patients with chronic diseases [16], which may help the patients quit smoking or at least increase their likelihood of quitting. Previous clinical trials have shown that brief cessation advice based on the AWARD (Ask, Warn, Advise, Refer and Do‐it‐again) model was effective in helping smokers quit smoking [16, 35]. Such brief cessation advice can be given within a minute, which is feasible to do in routine clinical practice, even by nurses with minimal training. In addition, as in many countries, in Hong Kong, the fast-paced and high-intensity nature of clinical settings may present a significant barrier to the delivery of smoking cessation counselling to patients with chronic diseases [33]. Health care resources commonly listed lack of time and resources as the reason for neglecting discussions pertaining to smoking cessation during counselling sessions [36]. Given these issues, in addition to the effort from health care professionals, nurturing a supportive macro environment for promoting smoking-free communities is critical. Public education should be strengthened to foster a community-wide perception change on smoking with correct information regarding smoking and smoking cessation, with the aim of putting pressure on the government to achieve tobacco endgame policies.

Of note, this study was conducted during the COVID-19 pandemic. Our results showed that the participants did not change their smoking behaviours or their motivation to quit during the pandemic. Instead, they admitted that smoking soothed negative emotions caused by their worries regarding their health condition and other issues in daily life. This notion is consistent with the findings of previous studies that smoking acts as a coping mechanism for patients with chronic diseases when they suffer psychological distress owing to their poor health conditions [8, 37]. Recent studies have proven that smoking increases the risk of contracting mild to severe COVID-19 and that patients with chronic diseases diagnosed with COVID-19 have a higher risk of experiencing severe symptoms [38] and mortality than those who do not have any chronic diseases [39]; hence, further efforts are required by nurses to promote smoking cessation among patients with chronic diseases, especially during this critical period.

### Limitations

This study has a number of limitations. First, all participants were from the same hospital; this might reduce the generalisability of our findings. Second, a skewed sample toward males may influence our findings due to gender differences. Third, the self-reporting approach in qualitative research implied that the result might have been subject to recall bias. Fourth, our findings do not show how participants’ demographic and clinical characteristics affected their risk perception, behaviours, attitudes, and smoking/smoking cessation-related experiences.

## Conclusions

This study has addressed a gap in the literature by soliciting smokers with chronic diseases’ perspectives and experiences regarding smoking/smoking cessation and their associated health issues. The deficit of knowledge among smokers with chronic diseases warrants the reinforcement of health education targeting this population. Appropriate advanced training is thus essential for nurses, who play a prominent role in raising the issue of smoking cessation with patients, to learn and implement advanced practices in smoking cessation interventions. This will help promote good health and minimise morbidity/mortality among smokers with chronic diseases.

## Supporting information

S1 Appendix EQUATOR research checklist: Consolidated criteria for reporting qualitative studies (COREQ): 32-item checklist. (DOCX)
